# Training and validation of a knowledge-based dose-volume histogram predictive model in the optimisation of intensity-modulated proton and volumetric modulated arc photon plans for pleural mesothelioma patients

**DOI:** 10.1186/s13014-022-02119-x

**Published:** 2022-08-26

**Authors:** Davide Franceschini, Luca Cozzi, Antonella Fogliata, Beatrice Marini, Luciana Di Cristina, Luca Dominici, Ruggero Spoto, Ciro Franzese, Pierina Navarria, Tiziana Comito, Giacomo Reggiori, Stefano Tomatis, Marta Scorsetti

**Affiliations:** 1 Radiotherapy and Radiosurgery Department, Humanitas Clinical and Research Center, IRCSS, Via Manzoni 56, 20089 Milan-Rozzano, Italy; 2 Department of Biomedical Sciences, Humanitas University, Milan-Rozzano, Italy

**Keywords:** Intensity-modulated proton therapy, Volumetric modulated arc therapy, RapidPlan, Knowledge-based planning, Pleural mesothelioma, Machine learning

## Abstract

**Background:**

To investigate the performance of a narrow-scope knowledge-based RapidPlan (RP) model for optimisation of intensity-modulated proton therapy (IMPT) and volumetric modulated arc therapy (VMAT) plans applied to patients with pleural mesothelioma. Second, estimate the potential benefit of IMPT versus VMAT for this class of patients.

**Methods:**

A cohort of 82 patients was retrospectively selected; 60 were used to "train" a dose-volume histogram predictive model; the remaining 22 provided independent validation. The performance of the RP models was benchmarked, comparing predicted versus achieved mean and near-to-maximum dose for all organs at risk (OARs) in the training set and by quantitative assessment of some dose-volume metrics in the comparison of the validation RP-based data versus the manually optimised training datasets. Treatment plans were designed for a prescription dose of 44 Gy in 22 fractions (proton doses account for a fixed relative biological effectiveness RBE = 1.1).

**Results:**

Training and validation RP-based plans resulted dosimetrically similar for both VMAT and IMPT groups, and the clinical planning aims were met for all structures. The IMPT plans outperformed the VMAT ones for all OARs for the contra-lateral and the mean and low dose regions for the ipsilateral OARs. Concerning the prediction performance of the RP models, the linear regression for the near-to-maximum dose resulted in D_achieved_ = 1.03D_predicted_ + 0.58 and D_achieved_ = 1.02D_predicted_ + 1.46 for VMAT and IMPT, respectively. For the mean dose it resulted: D_achieved_ = 0.99D_predicted_ + 0.34 and D_achieved_ = 1.05D_predicted_ + 0.27 respectively. In both cases, the linear correlation between prediction and achievement is granted with an angular coefficient deviating from unity for less than 5%. Concerning the dosimetric comparison between manual plans in the training cohort and RP-based plans in the validation cohort, no clinical differences were observed for the target volumes in both the VMAT and IMPT groups. Similar consistency was observed for the dose-volume metrics analysed for the OAR. This proves the possibility of achieving the same quality of plans with manual procedures (the training set) or with automated RP-based methods (the validation set).

**Conclusion:**

Two models were trained and validated for VMAT and IMPT plans for pleural mesothelioma. The RP model performance resulted satisfactory as measured by the agreement between predicted and achieved (after full optimisation) dose-volume metrics. The IMPT plans outperformed the VMAT plans for all the OARs (with different intensities for contra- or ipsilateral structures). RP-based planning enabled the automation of part of the optimisation and the harmonisation of the dose-volume results between training and validation. The IMPT data showed a systematic significant dosimetric advantage over VMAT. In general, using an RP-based approach can simplify the optimisation workflow in these complex treatment indications without impacting the quality of plans.

## Background

Malignant pleural mesothelioma (MPM) is an aggressive and refractory disease with a dismal prognosis of 6–8 months without treatment [1]. A trimodality approach, including various combinations of surgery, chemotherapy (CT) and radiotherapy (RT), is generally proposed to fit patients, with median survival ranging between 17 and 35 months and 5-year survival of 15% to 20% [2–6]. However, the evidence supporting such aggressive treatment is lacking, mainly due to the difficulties in designing and conducting prospective randomised trials in this rare disease. The role of RT is particularly controversial. Theoretically, RT should sterilise the pleural cavity after macroscopic surgical intervention to reduce the risk of local relapse. This role has become even more important in recent years, due to a significant shift in the surgical approach, from extrapleural pneumonectomy (EPP) to lung-sparing surgery, such as pleurectomy/decortication (P/D) and extended P/D [7, 8].

In addition to the already present critical organs in the thoracic area, the presence of an intact lung increases the difficulty in balancing the efficacy and toxicity of treatment. The ASCO guidelines recommend that hemithoracic adjuvant intensity-modulated radiation therapy may be offered to patients who undergo lung-sparing surgery but only in highly experienced centres, preferably in the context of a clinical trial [9].

Intensity-modulated RT (IMRT) and, more recently, Volumetric Modulated Arc Therapy (VMAT) have been applied in this clinical scenario with acceptable outcomes [10, 11]. Dosimetric comparisons showed a theoretical advantage of VMAT over IMRT [12, 13]. A population-based investigation demonstrated that in the USA, IMRT is currently the most common RT technique used to treat MPM [14]. Patel [15] reviewed the clinical studies evaluating the safety and efficacy of IMRT and concluded that it could be considered a viable option in patients with adequate survival expectations at the price of a few higher-grade toxicities.

Given the difficulties in finding a favourable balance between efficacy and side effects in MPM after lung-sparing surgery, proton therapy (PT), particularly in the form of intensity-modulated PT (IMPT), could represent an ideal solution. Indeed, the physical properties of protons can be exploited for a better sparing of normal tissues when treating the pleura, both in the postpneumonectomy setting and the lung-intact setting.

The consensus statement on proton therapy in mesothelioma by the International Particle Therapy Cooperative Group (PTCOG) [16] outlined the potential and the challenges of IMPT. This review confirmed how protons, compared to photon-based techniques, could contribute to a relevant reduction of the dose to the contralateral lung (associated with mortality and morbidity risks in mesothelioma patients) and most of the organs at risk (OAR) lime heart, liver and kidneys. The clear dosimetric advantage suggests that IMPT should be strongly considered as a viable solution. Some dosimetric studies showed a significant dose reduction in almost all OARs using PT [17–19]. Lorentini compared adjuvant IMRT and IMPT, showing the most significant advantages for the liver, ipsilateral kidney and contralateral lung while achieving improved coverage and target volume conformality [18].

Among the various factors contributing to the complexity of the RT process for mesothelioma patients (for both photons and protons), the treatment planning-related issues (target shape and extension, number and position of the OARs, dose-limiting constraints) were leading to suboptimal treatments [16]. The use of knowledge-based planning (KBP) might contribute to harmonising the plans' quality among different patients and streamline (and possibly automate) the inverse planning phase. The solution investigated in this study is the RapidPlan system (RP, Varian Medical Systems, Palo Alto, California, USA), a machine-learning-based and semi-automated planning method [20–22]. It aims to generate individualised optimisation constraints based on predicting the achievable dose distribution for any given patient and beam geometry. RP generates the individual constraints at the lower side of the uncertainty band of the predicted dose-volume histograms (DVH). The automated generation of the optimisation constraints should allow the inverse planning process without the need for interactive interventions (to create or adjust the dose-volume constraints and to guide the optimisation engine). Ultimately this should result in producing a clinically acceptable and high-quality plan.

Dumane [23] investigated the role of RP for MPM patients for VMAT showing improved sparing of the various OARs with reduced treatment planning time compared to manual procedures.

Delaney [24, 25] investigated the role of RP for protons in head and neck patients. Cozzi [26] studied RP for protons in patients with advanced hepatocellular carcinomas and compared these plans with VMAT plans and Celik for gastro-oesophagal cancer patients [27].

The present study aimed primarily to provide evidence of the usability of the RP KBP system for proton and photon planning by applying it to complex target volume structures and a large number of OARs. The specificity of MPM is given by the large size of the target, the number and the diverse sensitivity of the OARs involved. Although in principle the usability of KBP methods in MPM could be inferred from other treatment sites, the only study existing so far [23] was based on an older version of the system, including a dose calculation engine not accounting for full charged particle transport, all factors that might impact on the transferability of the results. Concerning IMPT, this study is, to date, the first attempt to investigate the role of RP-based planning with protons in MPM, the existing experience in head and neck and liver (relatively smaller targets and simpler anatomies (liver) or well-consolidated techniques (head and neck) do not allow to extend the results to MPM automatically. The performance of the RP model was benchmarked against manual optimisation. As a secondary aim, the project allowed quantifying, on a large cohort of patients, the relative figure of merit of IMPT versus VMAT and measuring the potential benefit, in terms of dose-volume sparing, for protons.

## Materials and methods

### Patient selection, contouring, and dose prescription

Eighty-two consecutive patients affected by MPM and treated with VMAT after lung-sparing surgery between 2012 and 2020 were included in this retrospective in-silico study. The study was approved by notification from the institutional ethical review board, and all patients provided consent to data analysis at the time of admission to the hospital.

Details about simulation, contouring, planning and delivery of the treatment have been previously published [11]. All patients were planned on computed tomography scans in the supine position. A thermoplastic thoracic mask was used for immobilisation. The clinical target volume (CTV) was delineated from the lung apex to the upper abdomen to include all pleural surfaces. Thoracotomy scars and draining scars were also included in the CTV. The planning target volume (PTV) was obtained with an isotropic expansion of the CTV of 5 mm.

The dose prescription was set to 44 Gy in 2 Gy per fraction as per the institutional practice [11], and all plans for the comparative study were normalised to the mean dose to the CTV.

The clinical aim to be achieved in the planning process for the CTV coverage and the PTV was D_95%_ ≥ 41.8 Gy (corresponding to 95% of the dose prescription). The aims for the OARs at risk were defined according to institutional practice and agreed with the QUANTEC recommendations as described in [11]. For the contralateral lung: D_mean_ ≤ 7 Gy, V_5Gy_ ≤ 60% (not explicitly expressed in [11] but part of the institutional clinical practice) and V_20Gy_ ≤ 7%. For the bowels, D_3cm3_ ≤ 50 Gy; for the Esophagus D_mean_ ≤ 34 Gy; for the heart, D_mean_ ≤ 30 Gy or V_30Gy_ ≤ 50%; for the kidneys V_18Gy_ ≤ 50%, for the liver D_mean_ ≤ 30 Gy and/or V_30Gy_ ≤ 50%; for the spinal canal D_01cm3_ ≤ 40 Gy; for the stomach D_mean_ ≤ 30 Gy. When not explicitly constrained, the mean dose was to be minimised in all cases. No specific constraints were applied for the ipsilateral lung. The same aims were considered valid for photon and proton plans.

### Treatment planning

The Eclipse treatment planning system (Varian Medical Systems, Palo Alto, USA) with the clinically released version 16.0 was used for all the patients and algorithms.

VMAT plans, in the RapidArc (RA) form, were optimised for 6 MV flattening filter-free beams from a Truebeam linac. According to the laterality of the target, as a class solution, three partial arcs (extending from about  −20° from the medial line to 180°). Collimator angles were 10°, 350° and 90° degrees. Minor adjustments of the arc length or collimator angles were applied to fit the anatomical characteristics of each patient best. The Photon Optimiser engine was applied for the inverse planning while the Acuros-XB algorithm [28] for the final dose calculation (with a matrix of 2.5 × 2.5 x 2.5 mm^3^).

IMPT plans were created using pencil beam spot scanning from the ProBeam proton system (Varian Medical Systems, Palo Alto, USA). The dose distribution was optimised using the fluence-based nonlinear universal Proton Optimizer (NUPO) [29]. The multifield simultaneous spot optimisation method was selected for all plans. The Proton Convolution Superposition algorithm was used for the final dose calculation with a grid of 2.5 × 2.5 x 2.5 mm^3^. A constant relative biological effectiveness (RBE) of 1.1 was applied. For notational simplicity, the same unit of measure (Gy) was used for photon and proton data, noting that protons have been scaled for the RBE.

All patients in the study were planned with a class solution geometry defined by two oblique fields with gantry angles set to 50° and 140° for the left-sided and the corresponding symmetric angles for the right-sided patients. Similarly to the photon case, minor adjustments of the gantry angles were allowed to fine-tune them according to the patients' anatomy. The robust optimisation method enabled the CTV to account for setup and range uncertainties considering ± 3 mm shifts in the isocentre along the x–y–z coordinates and ± 3% in the beam range. The 3 mm shifts are not intended as a proton-specific margin to the CTV but as positioning uncertainty. The robust optimisation should result in plans minimising the trade-offs derived from the applied uncertainties.

### The RapidPlan predictive model

The RP knowledge-based planning engine foundation consists of three pillars: (1) a model training environment, (2) a DVH prediction environment, and (3) the generation of personalised dose-volume constraints for the plan optimisation. The training phase is different for protons and photons due to the different characteristics of the dose patterns regarding the position of the OARs. Details about the specific implementation are provided in earlier studies [20, 21, 24, 25]. The second pillar aims to personalise and automate the inverse planning process by predicting, per each organ, the corresponding DVH (dosimetrically "achievable" as derived from geometrical relations between the beam arrangements and the specific anatomy of the patients and from the statistical model from the training process) which from the model). The third pillar aims to define personalised dose-volume constraints specific for each patient.

To derive from predicted DVH the actual dose-volume constraints to be used in the inverse planning process, it is necessary to create and store together with the model itself a set of general optimisation objectives per each of the OARs included in the model. The actual prospective objectives for any new patient will be derived from the list in the model and adapted to the DVH predictions. The objectives that users can define within the RP system are upper, mean, generalised equivalent uniform dose (gEUD), and line-type. For each of these, the defining parameters (e.g. the volume or dose values) and the priorities can be set explicitly or left to the prediction engine to place them below the inferior limit of the uncertainty band of the estimated DVH (the "generate" mode). Table 1 shows the list of general objectives defined for both the proton and photon models. Note that this list would be editable at any time by qualified users and adjustable according to any clinical need.

**Table 1:** CTV, PTV and OARs objectives implementation in the RapidPlan model

Structure	Constraint type	Volume	Dose	Priority
PTV and CTV	Upper	0%	101%	Generated
	Lower	100%	99.0%	Generated
Lungs	Upper	20%	Generated	Generated
	Mean	–	Generated	Generated
	Line	Generated	Generated	Generated
Breasts	Upper gEUD	35.0 (#)	Generated	Generated
	Upper gEUD	1.0 (#)	Generated	Generated
	line	Generated	Generated	Generated
Heart	Upper gEUD	35.0 (#)	Generated	Generated
	Upper gEUD	1.0 (#)	Generated	Generated
	line	Generated	Generated	Generated
Oesophagus	Upper gEUD	35.0 (#)	Generated	Generated
	Upper gEUD	1.0 (#)	Generated	Generated
	line	Generated	Generated	Generated
Stomach	Upper gEUD	35.0 (#)	Generated	Generated
	Upper gEUD	1.0 (#)	Generated	Generated
	line	Generated	Generated	Generated
Bowel bag	Upper gEUD	35.0 (#)	Generated	Generated
	Upper gEUD	1.0 (#)	Generated	Generated
	line	Generated	Generated	Generated
Kidneys	Upper gEUD	35.0 (#)	Generated	Generated
	Upper gEUD	1.0 (#)	Generated	Generated
	line	Generated	Generated	Generated
Spinal cord	Upper gEUD	35.0 (#)	Generated	Generated
	Upper gEUD	1.0 (#)	Generated	Generated
	line	Generated	Generated	Generated
Liver	Upper gEUD	35.0 (#)	Generated	Generated
	Upper gEUD	1.0 (#)	Generated	Generated
	Upper gEUD	Generated	Generated	Generated
Spleen	Upper gEUD	35.0 (#)	Generated	Generated
	Upper gEUD	1.0 (#)	Generated	Generated
	line	Generated	Generated	Generated

Heart, breasts, stomach, kidneys, liver and spleen were modelled and trained separately if ipsi- or if contra-lateral. For the lungs, only the contralateral volume was considered

CTV: clinical target volume; PTV: planning target volume; gEUD: generalized equivalent uniform dose; (#) the α parameter of gEUD; α  = 1 correspond to the mean dose while α  = 35 acts on the high dose region, in proximity of the near-to-maximum dose

The RP models investigated in this project were trained on a set of manually optimised IMPT and RA plans. The training was performed on a cohort of 60 patients. The plans, designed by experienced planners, were assessed according to the dose-volume planning aims listed above and the principle of dose minimisation to all structures without compromising the target coverage. Plans were included in the training set only if considered adequate from a clinical and physical perspective. This method aimed to minimise the variance due to plan quality. At the same time, the consistency of the sample should mitigate inter-patient variability compared to the minimal requirements of the model training (20 instances per OAR).

An independent cohort of 22 patients was used for an open-loop validation. IMPT and RA plans were optimised for each case using the RP method.

The split between training and validation cohorts was done by balancing the number of left and right cases and including a minimum of 20 female patients in the training group (to allow model training for the breasts). No other selection criteria were applied to split the samples.

In the training cohort, 40 patients were male and 20 females (17 and 5 respectively in the validation) and 28 patients were left-sided and 32 right-sided (9 and 13 respectively in the validation group).

The performance of the RP model was firstly measured by comparing predicted and achieved mean and near-to-maximum doses for each OAR and each patient in the validation cohort. In addition, a standard DVH comparison was performed among the four groups of plans (IMPT vs RA and training vs validation) to obtain a qualitative and quantitative assessment of the dosimetric quality of the RP-based plans for both techniques.

### Quantitative assessment of dose-volume metrics

The predictive performance of the RP model was measured by comparing and fitting some predicted vs achieved (after full optimisation) dose-volume metrics per each OAR. The metrics chosen were the mean dose and the near-to-maximum since they represent clinically relevant parameters (e.g. for parallel or serial organs). A highly performant RP model should result in a linear correlation between predicted and achieved metrics with a slope near 1. Quantitative metrics were derived from the DVH and included the mean dose and a variety of D_x_ and V_x_ parameters, with D_x_ representing the minimum dose that covers an x fraction of volume (in % or cm^3^) and V_x_ representing the volume receiving at least an x level of dose (in % or Gy). All parameters could be expressed in absolute (Gy or cm^3^) or relative (%) terms. The average DVHs were computed, for each structure and each cohort, with a dose binning resolution of 0.02 Gy (RBE). All the doses in the study are reported as Gy, i.e. in Cobalt Gray equivalent (corrected for the RBE factor).

The Wilcoxon matched-pairs signed-rank test was applied to evaluate the significance of the observed differences per each couple of plans. The threshold for statistical significance was *p* < 0.05.

## Results

**Fig. 1:**
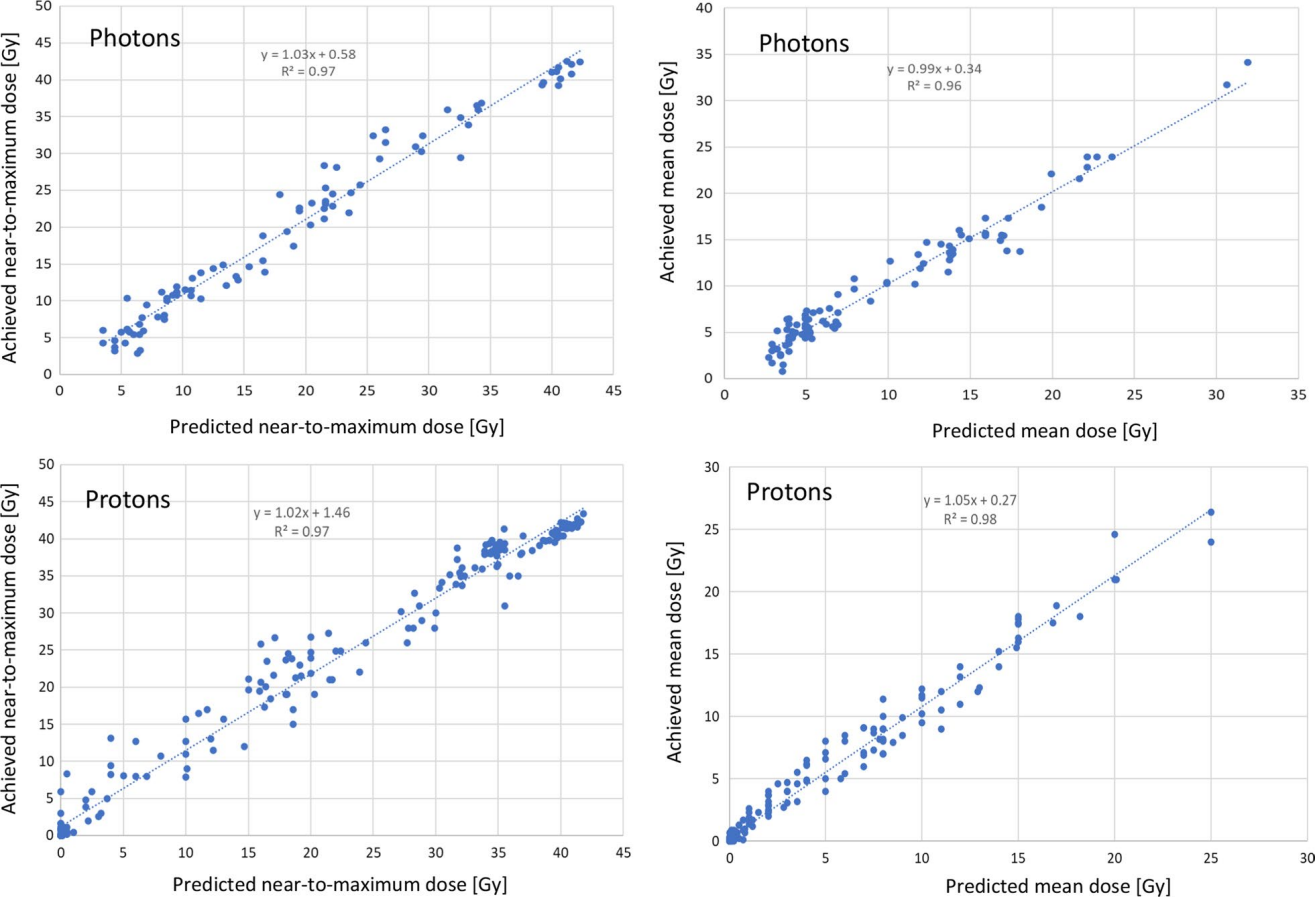
Scatter plots for the achieved vs predicted near-to-maximum and mean doses for the proton and photon plans in the validation cohort. The linear regression trends are overlaid to the data with the fit results

The predictive performance of the proton and photon models was summarised in Fig. 1, where the model-predicted mean and near-to-maximum (D_1%_) doses for the various OARs were compared to the achieved ones after the full optimisation and dose computation phases. The data are presented separately for the photon and the proton plans and are only relative to the patients in the validation cohort. The linear regression fit coefficient ranged from 0.96 to 0.98, while the slope of the linear trend ranged from 0.99 to 1.05 for the mean dose for the VMAT or IMPT plans, respectively and from 1.03 to 1.02 for the near-to maximum doses.

**Fig. 2:**
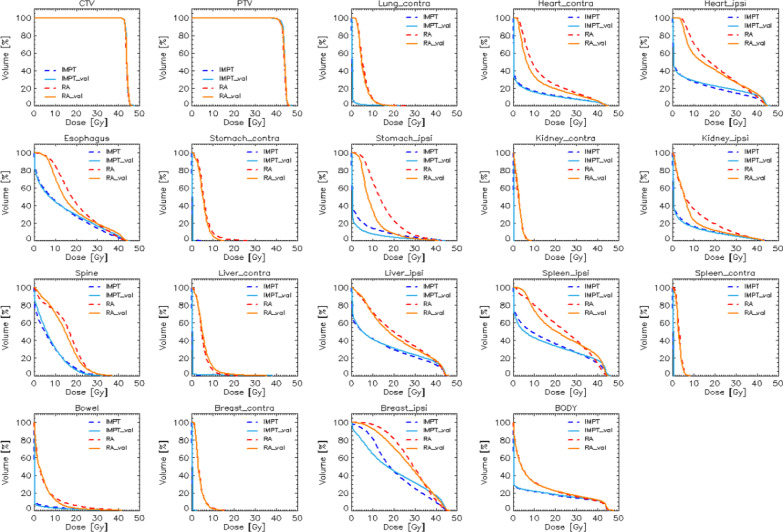
average dose-volume histogram comparison for all the target and organ at risk structures for the proton and photon plans and the training (dashed lines) or validation (solid lines) cohorts

Figure 2 shows the average DVHs for the targets and all the OARs for the IMPT and RA plans. The training (manually optimised) data are represented as dashed lines, while the validation (RP-based optimisations) are presented as solid lines. From a qualitative perspective, the RP-based plans from the validation cohort resulted in equivalent or better to the manually optimised plan for the training cohort. As expected from prime principle considerations, the OAR sparing resulted quantitatively better for IMPT in all the cases. A complete sparing was achieved for contralateral structures. At the same time, in the case of ipsilateral organs, the IMPT allowed a large sparing in the medium to low dose ranges while resulting in similar near-to-maximum doses for the ipsilateral structures.

Table 2 summarises the quantitative analysis of the DVHs for the CTV, PTV and healthy tissue, while Table 3 provides the findings for all the OARs. The columns in the table refer to the RA or IMPT plans and are stratified for the manually optimised plans or the RP-based plans for the training and validation cohorts of patients. The clinical planning goals are reported for each parameter. Data are presented as mean values with interpatient variability reported as on standard deviation of the mean. The statistical significance of the observed difference is reported if significant for any given couple of plans.

**Table 2:** Summary of quantitative DVH analysis for the CTV, PTV and healthy tissue

	Aim	RA_training	RA_validat	IMPT_training	IMPT_validat	p
*CTV*						
Mean [Gy]	44.0	44.0 ± 0.0	44.0 ± 0.0	44.0 ± 0.0	44.0 ± 0.0	–
D_95%_ [Gy]	≥ 41.8 (95%)	43.0 ± 0.2	42.9 ± 0.2	43.1 ± 0.2	43.0 ± 0.3	–
D_1%_ [Gy	Minim	45.5 ± 0.2	45.8 ± 0.3	45.7 ± 0.4	46.0 ± 0.5	AB
CI [%]	Minim	4.7 ± 0.8	5.2 ± 0.8	4.4 ± 1.0	4.6 ± 1.2	ABCD
*PTV*						
Mean [Gy]	44.0	43.7 ± 0.1	43.8 ± 0.1	43.9 ± 0.1	43.9 ± 0.1	BD
D_95%_ [Gy]	≥ 41.8 (95%)	41.8 ± 0.6	42.0 ± 0.5	42.6 ± 0.4	42.5 ± 0.5	BD
D_1%_ [Gy]	Minim	45.6 ± 0.2	45.8 ± 0.3	45.8 ± 0.3	45.9 ± 0.4	AB
*Healthy tissue*						
Mean [Gy]	Minim	11.3 ± 2.3	11.5 ± 1.7	7.6 ± 1.7	7.9 ± 1.5	ABC
V_5Gy_ [%]	Minim	45.6 ± 9.4	44.0 ± 6.3	23.7 ± 4.9	24.0 ± 3.7	BC
V_10Gy_ [%]	Minim	32.3 ± 6.7	32.0 ± 4.5	21.6 ± 4.5	21.9 ± 3.5	BC

Data are reported for the training and validation cohorts and for the RA and IMPT techniques. The validation plans were optimised using the RapidPlan model

CI = (D_5%_-D_95%_)/D_mean_ RA: RapidArc; IMPT: intensity modulated proton therapy; training: model training subset; validat: model validation subset. Statistical significance: a = IMPT_training vs IMPT_validat; b = IMPT_training vs RA_training; c = IMPT_validat vs RA_validat; d = RA_training vs RA_validat

**Table 3:** Summary of quantitative DVH analysis for the OARs

	Aim	RA_training	RA_validaz	IMPT_training	IMPT_validaz	p
*Contralateral lung*						
Mean [Gy]	≤ 7	5.9 ± 1.1	5.5 ± 0.9	0.3 ± 0.4	0.3 ± 0.4	BC
V_5Gy_ [%]	≤ 60	53.9 ± 17.5	46.2 ± 13.6	1.4 ± 2.0	1.5 ± 2.0	BC
V_20Gy_ [%]	≤ 7	0.4 ± 0.8	0.2 ± 0.4	0.2 ± 0.6	0.2 ± 0.5	B
*Bowel*						
Mean [Gy]	minim	5.1 ± 3.5	4.6 ± 2.6	1.1 ± 1.4	0.8 ± 0.9	ABC
D_3cm3_ [Gy]	≤ 50 Gy	27.4 ± 12.4	27.4 ± 13.8	22.0 ± 16.8	19.8 ± 17.2	ABC
*Contralateral Breast*						
Mean [Gy]	Minim	3.7 ± 0.8	3.6 ± 0.8	0.1 ± 0.1	0.1 ± 0.1	BC
*Ipsilateral Breast*						
Mean [Gy]	Minim	28.8 ± 2.8	27.4 ± 5.4	20.8 ± 4.6	20.2 ± 10.9	BC
*Esophagus*						
Mean [Gy]	≤ 34	20.1 ± 4.8	18.3 ± 5.0	12.7 ± 6.5	13.1 ± 6.5	BC
D_1cm3_ [Gy]	minim	37.0 ± 5.8	38.8 ± 5.3	35.4 ± 8.5	37.7 ± 6.7	ABD
*Heart*						
Mean [Gy]	≤ 30	13.3 ± 3.8	14.7 ± 5.1	5.8 ± 2.6	7.1 ± 3.4	BC
V_30Gy_ [%]	≤ 50	14.3 ± 8.4	18.3 ± 10.7	8.7 ± 5.2	11.9 ± 7.0	BC
*Ipsilateral Kidney*						
Mean [Gy]	Minim	10.5 ± 6.7	8.6 ± 4.3	4.8 ± 4.5	4.6 ± 3.5	BC
V_18Gy_ [%]	≤ 50	10.8 ± 11.4	10.2 ± 8.9	21.8 ± 21.4	13.1 ± 10.5	BC
*Contralateral Kidney*						
Mean [Gy]	Minim	2.7 ± 1.2	2.7 ± 1.0	0.1 ± 0.1	0.1 ± 0.1	BC
*Ipsilateral Liver*						
Mean [Gy]	≤ 30	21.9 ± 5.3	21.1 ± 5.8	13.4 ± 3.7	14.1 ± 4.4	BC
V_30y_ [%]	≤ 50	33.5 ± 12.7	30.6 ± 12.7	21.2 ± 7.5	23.7 ± 9.4	BC
*Contralateral Liver*						
Mean [Gy]	≤ 30	5.1 ± 1.2	5.7 ± 0.5	0.1 ± 0.1	0.4 ± 0.4	BC
Spinal canal						
D_0.1cm3_ [Gy]	≤ 40	28.9 ± 6.0	30.6 ± 5.7	28.2 ± 6.4	27.7 ± 5.9	CD
*Ipsilateral Spleen*						
Mean [Gy]	Minim	24.1 ± 6.6	23.3 ± 6.5	15.7 ± 6.0	14.9 ± 5.0	BC
*Contralateral Spleen*						
Mean [Gy]	Minim	3.2 ± 0.8	2.9 ± 1.0	0.1 ± 0.1	0.1 ± 0.1	BC
*Ipsilateral Stomach*						
Mean [Gy]	≤ 30	15.0 ± 4.1	9.8 ± 3.1	4.3 ± 2.9	2.4 ± 2.3	BCD
*Contralateral Stomach*						
Mean [Gy]	≤ 30	6.1 ± 2.7	5.4 ± 1.9	0.1 ± 0.2	0.1 ± 0.1	BC

Data are reported for the training and validation cohorts and for the RA and IMPT techniques. The validation plans were optimised using the RapidPlan model

RA: RapidArc; IMPT: intensity modulated proton therapy; training: model training subset; validaz: model validation subset. Statistical significance: a = IMPT_training vs IMPT_validaz; b = IMPT_training vs RA_training; c = IMPT_validaz vs RA_validaz; d = RA_training vs RA_validaz

In general terms, RA and IMPT resulted equivalent for the CTV and PTV in both the training and validation cohorts, demonstrating the adequate coverage of the CTV as required by the study design. For the healthy tissue and all the OARs, the quantitative data confirm that IMPT findings are systematically better than the RA data. For some OARs, the average DVH graphs from Fig. 1 suggested an improvement in the validation cohort. Nevertheless, this did not result numerically or clinically remarkable for the various clinical aims reported in Table 3, with the possible exception of the ipsilateral stomach. As a caution note, the observed difference might be attributed to RP compared to manual methods, and the two cohorts included different patients.

## Discussion

Two RP models for IMPT and VMAT in the setting of adjuvant RT after lung-sparing surgery for MPM were defined, trained and validated. To our knowledge, this is the first study creating such models in this clinical scenario. Limited to VMAT, Dumane [23] reported the validation of an RP model trained on 57 patients and validated on 23 cases, i.e. on a sample size consistent with ours. The main difference with that study, besides the focus on photons only, relies on a different dose prescription (50.4 Gy in 1.8 Gy per fraction compared to 44 Gy in 2 Gy fractions) and the use of an older dose calculation algorithm not fully accounting for charged particle transport (the anisotropic analytical algorithm, AAA) compared to the type-c Acuros algorithm applied in our study. The algorithm difference might impact some metrics, particularly in the lung and the proximity of highly heterogeneous structures. In general, both studies, relatively to photons, demonstrate the advantage of RP over the manual practice.

The use of RP for proton therapy can bring several advantages. One is the improvement of planning effectiveness with more consistent results among different planners and patients. This is more relevant for tumour entities challenging in terms of the OARs sparing. Pleural mesothelioma is paradigmatic due to many OARs, the geometrical complexity and the tight constraints (particularly for the contralateral lung).

Concerning the harmonisation of the plan quality and the (partial) automation of the inverse planning process (automatic definition of the constraints and un-attended optimisation), both objectives were met as suggested by the results for VMAT and IMPT. The first is confirmed, e.g., by the small inter-patient variance of the dose-volume metrics for most OARs and the high correlation between predicted and achieved mean and near-to-maximum dose. It is essential to outline that the harmonisation aim is potentially prone to the characteristics of the population under investigation. Significant variations of the target volumes among the patients and, similarly, in the geometrical relation between targets and OARs can impact the plan quality. The RP models validated in this report would allow a safe and high-quality planning process for both VMAT and IMPT techniques. The results of the current study are consistent with the earlier published data in head and neck, liver and oesophagal cancer patients [24–27] and constitute a further step in the evidence generation (at the in-silico level) of the role of RP for both VMAT and IMPT for a, so far missing, tumour site.

Among the limiting factors of the RP investigation, the sample size is always a potential issue. In the present case, the male–female ratio was unbalanced due to the clinical population characteristics. To train the models for the breast structures (RP requires a minimum of 20 entries to train the model for any given OAR), it was therefore necessary to limit the number of female cases in the validation set to only 5. The sampling of all other structures results more appropriate, and in particular, the split of left–right cases was well balanced. The second limiting factor is the "scope" of the models. Only patients with lung preserving surgery were included in the study. This means that the applicability of these models to other surgical conditions should be specifically validated or, preferably, dedicated models should be trained.

As a secondary aim of the study, we also provided a dosimetric comparison of VMAT and IMPT. Our results confirm that protons better spare all OARs in the thoracic and abdominal regions. This is particularly relevant for the contralateral organs. The clinical impact of this better sparing is yet to be demonstrated; however, it is well known that contralateral lung dose is correlated with the risk of pneumonitis and death [30, 31]. Rice successfully treated ten patients as adjuvant or salvage treatment after P/D [32]. With a median prescribed dose of 55.0 Gy (Cobalt Gy equivalent), the two years local control was 90%, with a median survival of 19.5 months. No patient had toxicity higher than G2, neither acute nor late.

Pan from MD Anderson reported on four patients treated with IMPT with intact lungs. IMPT plans were compared with IMRT plans for the same patients, showing the superiority of protons [33]. Patients were successfully treated with no safety alert. Another available clinical series includes ten consecutive patients treated with IMPT in the presence of two intact lungs [34].

Although limited by the small sample size, these clinical experiences highlight the feasibility of IMPT, confirming the high expectancies for a clinical benefit using this technique in patients with MPM.

As being designed as an in-silico planning study, no direct translation of the dosimetric results into clinical outcomes can be done. However, because of the disease's rarity, our series is large and homogeneous, reflecting actual patients treated with VMAT. The innovative approach of the RP model in this clinical scenario is our major strength.

## Conclusion

Two models were successfully trained and validated for VMAT and IMPT plans for pleural mesothelioma. The performance of the two models resulted in a high concordance between predictions and achievement. The IMPT plans outperformed the VMAT plans for all the OARs (with different sparing potential for contra- or ipsilateral structures). RP-based planning and IMPT might lead to significant dosimetric advantage and workflow simplification in managing these complex treatment indications.

## Data Availability

Zenodo repository.
